# Moral conflicts from the justice and care perspectives of japanese nurses: a qualitative content analysis

**DOI:** 10.1186/s12910-023-00960-7

**Published:** 2023-10-04

**Authors:** Kayoko Tsunematsu, Atsushi Asai, Yasuhiro Kadooka

**Affiliations:** 1 Department of Bioethics, Faculty of Life Sciences, Kumamoto University, 1-1-1 Honjo, Chuo-ku, Kumamoto, 8608556 Japan; 2 Department of Medical Ethics, Tohoku University Graduate School of Medicine, 2-1 Seiryo-machi, Aoba-ku, Sendai, Miyagi 980-8575 Japan

**Keywords:** Care, Japan, Justice, Medical ethics, Moral conflict, nurse, Qualitative analysis

## Abstract

**Background:**

Healthcare professionals use the ethics of justice and care to construct moral reasoning. These ethics are conflicting in nature; different value systems and orders of justice and care are applied to the cause of actual moral conflict. We aim to clarify the structure and factors of healthcare professionals’ moral conflicts through the lens of justice and care to obtain suggestions for conflict resolutions.

**Method:**

Semi-structured interviews about experiences of moral conflict were conducted with Japanese nurses recruited using the snowball sampling method. Interviews were conducted based on the real-life moral conflict and choice interview. Interviews were recorded and transcribed verbatim, then analyzed based on the interpretive method of data analysis. Verbatim transcripts were read four times, first to get an overall sense of the conflict, then to understand the person’s thoughts and actions that explain the conflict, and third and fourth to identify perspectives of justice and care, respectively. Each moral perspective was classified into categories according to Chally’s taxonomy.

**Results:**

Among 31 responses, 2 that did not mention moral conflict were excluded, leaving 29 responses that were analyzed. These responses were classified into six cases with conflict between both justice and care perspectives or within one perspective, and into two cases without conflict between perspectives. The “rules” category of justice and the “welfare of others” category of care were included in many cases of conflict between two perspectives, and they frequently occurred in each perspective.

**Conclusions:**

The nurses in this study suggest that they make moral judgments based on moral values that are intertwined with justice and care perspectives complex manner.Organizational, professional, and patient-related factors influenced conflicts between justice and care. Additionally, multiple overlapping loyalties created conflicts within justice perspectives, and multifaceted aspects of care-provider’s responsibility and patient need created conflicts within care. Decision-making biased towards one perspective can be distorted. It is important to consider ethical issues from both perspectives to resolve conflicts, especially the effective use of the ethics of care is recommended.

**Supplementary Information:**

The online version contains supplementary material available at 10.1186/s12910-023-00960-7.

## Background

The debate over the ethics of justice and care began nearly 40 years ago and remains controversial. Gilligan proposed an ethic of care in opposition to the ethics of justice, on which conventional moral developmental theories are based, and outlined the perspective of justice and care as an alternative way of elucidating moral problems [1]. Early discussions of care ethics tended to remain confined to care relationships in the private sphere, starting with the mother-child relationship in the home and emphasizing the priority of care rather than justice [1–5]. Since then, the debate has turned toward meaningful relationships between justice and care, and care ethics has come to discuss its applicability to the public sphere, such as politics [2, 6–9].

The difference between traditional justice and an ethic of care is obvious when those perspectives are juxtaposed. The ethics of care emphasizes attentiveness, trust, responsiveness to needs, narrative nuances, and cultivating caring relations [5] and considers the contextual factors of moral issues while being aware of the maintenance of relationships and emotional connections between people involved in moral issues [8]. The ethics of justice emphasizes fairness, equality, individual rights, abstract principles, and their consistent application [5], and competing abstract moral principles in a moral problem are weighed up and a conclusion drawn [8]. These contrasting ethics allow the interpretation of the essential causes of moral problems in the medical field [10–15].

Healthcare professionals, regardless of the type of profession, use the perspective of justice and care to construct moral reasoning [16–18]. Since health care is best provided with respect for the patient’s wishes, the moral reasoning of the health care professional should be respectful of the patient’s needs and wishes. However, some studies of nurses’ use of justice and care perspectives have reported that individuals have different predominant perspectives, which in turn influences patient care practices [10–13]. Predominant perspectives lead individuals to ethical decisions that are justified from their respective perspectives. Such moral choices can be undesirable in terms of patient outcomes, and vice versa. For example, nurses with a predominantly care perspective are likely to be inactive in their job if they are unable to provide the nursing care that they believe will be most beneficial to their patients [12, 13]. Moreover, nurses with a predominantly justice perspective tend to be in managerial positions and have begun to value the bureaucratic advocate roles more, and as the pressure of administration increases, they are more likely to be active in nursing [12]. Therefore, a predominant perspective has benefits, like improvement of patient service quality and management, but if it is actually difficult to provide such services, it may make it difficult to continue working or place too much emphasizing management factors. Furthermore, care predominance tends to be seen in nurses with more clinical experience; because they have technical expertise in treatment and care, they tend to focus on individualized care, considering both perspectives [10]. Therefore, the predominance of perspectives and whether there is a single or multiple perspectives that can influence moral judgment varies with individuals, and as a result, the decisions that individuals make are not uniform.

The basic principles of justice and care have several differences. First, their moral concepts are different. The ethics of justice emphasize the moral concepts of rights and rules, whereas the ethics of care emphasizes the concepts of responsibility and relationships [1, 6]. Whereas the rights conception of morality is geared toward an objectively fair or just resolution to moral dilemmas upon which all rational persons could agree, the concept of responsibility focuses on the limitations of any particular resolution and is oriented to insistent contextual relativism [1].

Second, the level of abstraction of conflict resolution approaches is different. The ethics of justice respond formally to the application of abstract principles, while that of care a connection to concrete circumstances [1, 4, 6]. Principle-based approaches differ significantly from context-based ethics of care in that they fail to address events taking place in the specific context [4]. These approaches are contradictory in that they are formal in the ethics of justice and substantive in the ethics of care.

Third, the application of fairness is different. Fairness is a central concept in many ethical theories. The ethic of justice must be acceptable to every member of a society and is based on impartial consideration [19]. In contrast, the ethics of care is based on partial considerations applied to the specific others who have a relationship with the caregiver [20].

As stated above, justice and care ethics are conflicting in nature. To resolve this moral conflict, the integration of the two perspectives by incorporating one into the ethical framework of the other, or by prioritizing one perspective, has been discussed [5, 9, 19]. Moreover, in the healthcare field, the conflicts between justice and care causes moral conflict among healthcare professionals, and therefore, it is recommended to strike a balance between justice and care perspectives or an integrated approach for both perspectives adopted to resolve the conflict [21, 22]. However, conceptual discussion alone may not suffice to resolve actual moral conflicts. In one survey, nurses expressed integrated concerns from the perspectives of justice and care, but the implementation of decision-making based on them was constrained by differences in role authority among professionals and the scope of institutional work [23]. In addition, it is difficult for healthcare professionals to help every patient fairly while meeting the needs of a specific patient [24], and as they place greater emphasis on professional duties, they may become more concerned with performing clinical procedures on patients than responding to their specific needs [14]. Thus, the integration of the perspectives is impossible when the aims of the two perspectives are regarded as contradictory and mutually exclusive.

Different value systems and orders of justice and care perspectives are considered a cause of actual moral conflict [14, 15, 23]. Few empirical studies have clarified the actual aspect of moral conflicts from both perspectives, and the specific structure of such conflicts has not been clarified [11, 15]. To gain resolutions for moral conflicts, it is necessary to elucidate how the elements of the perspectives of justice and care constitute moral conflicts. Therefore, we need empirical knowledge of moral conflicts from the perspective of justice and care, that is, findings from empirical research. Such empirical research would provide concrete evidence that justice and care reasoning is utilized during the moral conflicts of healthcare professionals, and contribute to the development of judgment and conflict resolution strategies using justice and care perspectives. Therefore, we focused on nurses who have professional roles and obligations [25], who provide close and continuous daily care to patients [26], and who have elements of justice and care perspectives. Nurses, including midwives, constitute more than half of the healthcare workforce in many countries and thus play an important role in healthcare [27]. Therefore, this study aimed to clarify the structure and factors of the moral conflict between justice and care that healthcare professionals, mainly nurses, encountered and offer suggestions for resolving the conflict.

## Methods

### Taxonomies of justice and care

To clarify the perspectives of individual justice and care from the experiences of nurses’ moral conflict, we needed specific explanations based on the definitions of each perspective. Several studies have used Chally’s taxonomy [10] and Lyons’ coding schema [28] as criteria for identifying justice and care perspectives [11, 15, 16]. Analyzing complex and unclear ethical thinking in empirical research based on personal experience requires using various theoretical frameworks, concepts, and definitions, one of which is Chally’s taxonomy [29, 30]. This taxonomy was created by Chally to categorize the justice and care perspectives of the profession. Chally referred to Brown’s taxonomy of care, Gilligan’s and Rogers’ justice, which were developed for adolescent girls, and revised them to remove similar categories and fit the professional perspective [10]. Chally’s conceptions of justice are based on multiple perspectives, including professional ethics and ethical principles as well as deontology and utilitarianism. These may not be pure concepts of justice, but they consist of concepts necessary for the healthcare profession, such as legal regulation of health care delivery, patient rights, and the duties and commitments of the healthcare profession. We considered that Chally’s conceptions of care, based on traditional arguments of feminist origin, could be replaced by the relationship with patients in health care in that it responds to the needs of others in a particular relationship, despite the lack of fairness and nonconformity to the ethics of principles discussed in modern ethics. Therefore, we adopted Chally’s taxonomy of care, which is based on traditional Gilligan’s arguments.

In addition, Lyons’ coding scheme was used to roughly classify moral reasoning by perspective, but the specific concept of each perspective was not clarified [15, 28]. Chally verified that it was possible to identify and classify nurses’ justice and care perspectives using Chally’s taxonomy [11]. Therefore, we adopted Chally’s taxonomy to examine in detail the perspectives of justice and care that constitute moral conflict [10]. Table 1 illustrates Chally’s taxonomy.

**Table 1:** Chally’s Taxonomy of Justice and Care

Moral categories	
**Justice perspective**	
Roles	Roles of professionals and roles expectations
Rights	Making moral decisions based on a person’s rights; maintaining social order through fixed principles
Rules	Following orders or protocols and not thinking about the situation
Obligations and commitments	Commitment to the organization under obligations of the profession and the organization
Legal issues	Compliance of fixed rules and laws; the maintenance of social order through the legal system
Societal concerns	Concern for fairness; concern about the interests of the society
**Care perspective**	
Welfare of others	Present and future concerns about the welfare of the patients and families; responding to the specific needs of the patients and families, both physically and psychologically
Care of self	Self-protective function; taking pride in the quality of care given
Appreciation of differences	Attempts to understand others’ ways of behaving, their feelings, their thoughts, and their experiences
Not hurting	Protecting patients from pain and hurt; concerns about the pain that the patient endures
Attachment and connection	A relationship with patients based on love, acceptance, and responding to patients’ own wishes, as expressed to patients, family members, and colleagues

### Data collection

The inclusion criteria for this study were Japanese nurses who encountered moral conflicts in clinical settings. This study conducted semi-structured interviews with 31 nurses recruited using snowball sampling. The first author conducted the interview surveys between September 2011 and March 2013. Interviews were based on a “real-life moral conflict and choice interview” by Gilligan et al. [31]. After obtaining Gilligan’s permission, the Japanese version of the “real-life moral conflict and choice interview” (see Additional file 1) created after verification based on back-translation and pilot study was used as an interview guide. Using the guide, we systematically listened to the situation, the focus of the conflict, the moral choices and their reasons, and the thoughts and feelings at the time of the conflict. This method was developed to interpret the complex narratives of people in real-life moral conflicts and choices using 11 questions to systematically elicit and analyze the focus of moral conflicts, choices, and thoughts and feelings during conflicts. Following previous studies of nurses using this method, we conducted a preliminary study to confirm that this interview technique can be used to describe nurses’ ethical conflicts and interpret their ethical thinking. We analyzed each interview and then recruited the next subject. Finally, data collection was completed when the subjects’ genders were balanced and trends in justice and care categories and the structures of conflict in both perspectives had been identified.

### Data analysis

Based on the premise that nurses’ moral conflicts are due to the conflict between justice and care perspectives [10–15], this study employed a method of listening to narratives from the perspective of justice and care. Therefore, by applying the narratives of each nurse to the classification of justice and care and analyzing them, we considered it possible to clarify what kinds of viewpoints of justice and care create moral conflict in practice. The “interpretive method of data analysis” of Brown et al. [31, 32] is a method to interpreting Gilligan’s interview guide [32]. This interpretive method was used to analyze the data after participants’ responses were recorded and transcribed verbatim (see Additional file 2). Using to this method, the first author read the verbatim transcript four times. The verbatim transcript was read first to get an overall sense of the conflict, then to understand the person’s thoughts and actions that explain the conflict, a third time to identify concepts related to the care perspective, and then fourth to identify concepts related to the perspective of justice. After four readings, the first author summarized the moral conflicts and action options in each case and extracted the reasoning for each action from the perspectives of justice and care. Each moral perspective was classified into categories according to Chally’s taxonomy [10] (Table 1). The justice perspective is constructed in six categories and concerned with issues of inequality and strongly values the idea of reciprocal rights and respect for individuals [10]: roles, rights, rules, obligations and commitments, legal issues, and societal concerns. The care perspective comprises five categories and concerned with issues of attachment and strongly values attention and response to need [10]: welfare of others, care of self, appreciation of differences, not hurting, and attachments and connections. This series of classifications was repeated until all authors agreed.

### Ethical considerations

This research was approved by the ethics committee of Kumamoto University Faculty of Life Sciences (approval number: 1228). All interviews were conducted after obtaining written informed consent from participants.

## Results

### Demographics

The consent rate for research participation was 100% among the 31 nurses who were recruited for the research. Of them, 2 participants who did not mention moral conflict were excluded, and 29 participants’ responses were analyzed: 13 men and 16 women with a mean age of 35 years (26–45) and mean clinical experience of 10 years (4–11). In all, 4 participants had a master’s degree (13.8%), 8 a bachelor’s degree (27.6%), 6 an associate degree (20.7%), and 11 a nursing diploma (37.9%). By position, 24 were active nurses (82.8%), 2 worked as nurse manager, 1 as assistant nurse manager, and 21 as staff nurses. Of the active nurses, six worked in internal medicine, five in psychiatry, three in pediatrics, two in surgery, three in ophthalmology, two in emergency, two in intensive care units, and one in recovery rehabilitation. The mean interview time was 34 min (23–60).

### Conflict scenes, presence of moral perspectives, and conflicts between moral categories

Table 2 shows a summary of 29 moral conflicts narrated by participants each with its moral perspective and moral category. Of the 29 cases, cases with conflict between both justice and care perspectives or between one perspective were classified into six cases, and cases without conflict between their perspectives were classified into two, thus yielding eight types.

**Table 2:** Presence and Conflict of Moral Perspectives and Moral Categories of Justice and Care

Case No	Scene	Moral conflict	Option	Justice perspective	Care perspective
1. Conflict between the justice and care moral categories					
C1	A physician decided to hospitalize an elderly patient who had refused to be hospitalized at the request of the family.	Whether to oppose physicians’ decision to hospitalize patients according to family’s request	Oppose		**(Welfare of Others)** Hospitalization is stressful for the patient
			Do not oppose	**(Roles)** Follow the direction of the physician who has the authority to decide the hospitalization of the patient **(Obligation and commitments)** Fulfill the obligation to act under the direction of the physician in the hospital organization	
C2	During surgery, the work hours of the scrub nurse were coming to an end.	Whether to ask another staff member to take over the task of delivering surgical instruments	Ask	**(Rules)** Observe the rules of the ward that a person does not work overtime **(Obligation and commitments)** Do not disrupt organization control	
			Do not ask		**(Welfare of Others)** Avoid the risk to the patient due to the change of surgical instrument delivery role
C3	After cataract surgery in a hepatitis C patient, the patient’s head was contaminated with postoperative drainage.	Whether to use gloves to wipe off postoperative drainage on the patient’s head	Use gloves	**(Rules)** Act according to infection prevention measures	
			Do not use gloves		**(Welfare of Others)** Immediately wiping the effluent makes the patient comfortable
C4	Because the patient died after ambulance transport, postmortem care for a deceased patient had to be provided.	Whether to perform the postmortem care on a dead patient with his family	Perform with family		**(Welfare of Others)** Bereaved family can organize their feelings **(Care of self)** Act in line with belief in building a trusted relationship with patients
			Do not perform with family	**(Rules)** Prioritize other duties	
2. Conflict between combinations of justice and care moral categories					
C5	An elderly patient with an ileus tube had been physically restrained	Whether to continue physical restraint to prevent extubating of the ileus tube	Continue	**(Rules)** Carry out other duties	**(Welfare of Others)** Prevent physical pain caused by removal of the ileus tube
			Do not continue	**(Rights)** Speak for the patient’s desire to remove the restraint band **(Rights)** Protect the human rights of patients **(Societal concerns)** Put yourself in patient’s shoes	**(Care of self)** Dispel feeling of remorse for not being able to comfort the patient **(Not hurting)** Protect patient dignity
C6	An elderly terminally ill patient became more restless every night.	Whether to suggest increased sedation for the terminally ill patient to physicians	Suggest	**(Rules)** Prioritize other tasks over dealing with the restless patient	**(Welfare of Others)** Weak sedative effects make patients more restless
			Do not suggest	**(Rights)** Respect the patient’s right to self-determination about receiving sedation **(Societal concerns)** Put yourself in patient’s shoes	**(Welfare of Others)** The patient can live comfortably for the rest of their lives
C7	A terminally ill patient with dysphagia wanted oral intake, but his family was against oral intake.	Whether terminally ill patients at risk of aspiration should be given oral intake	Take orally	**(Rights)** Respect the patient’s wishes for oral intake **(Societal concerns)** Put yourself in patient’s shoes	**(Welfare of Others**) Relieve patient’s thirst and hunger
			Do not take orally	**(Roles)** Physician’s expert opinion is required to determine oral intake permission	**(Appreciation of differences)** Understanding the family’s wishes for the patient’s survival
C8	Chest compressions continued for a patient unlikely to be resuscitated until the family arrived.	Whether to oppose the implementation of slow codes by physicians and senior nurses	Oppose	**(Rules)** Treat the patient’s corpse with care	**(Welfare of Others)** Stopping cardiopulmonary resuscitation in dead patients is best
			Do not oppose	**(Roles)** Follow the judgment of physicians and senior nurses about slow code continuation	**(Appreciation of differences)** Decisions made by physicians and senior nurses are for the sake of the family
C9	One of the two patients in charge of an intensive care unit nurse died.	Whether to see off a deceased patient in charge	See off	**(Roles)** Seeing off is the job of the healthcare provider **(Rules)** Follow the custom of seeing off a deceased patient experienced at a previous workplace	**(Welfare of others)** Bereaved families need emotional support **(Attachment and connection)** I want to snuggle patients slowly after they die
			Do not see off	**(Roles)** Save the life of another patient with a turn for the worse **(Rules)** Follow the absolute instructions of the team leader on the shift about the lifesaving of other patients	**(Appreciation of differences)** Understand the burden on the colleagues who represent critically ill patients
3. Conflict between the combination of justice and care moral categories and justice categories					
C10	A patient who vomited blood at night was raced to hospital, and the patient needed a blood transfusion	Whether to voluntarily obtain consent for blood transfusion from the patient’s family without a physician’s order	Obtain consent	**(Rules)** Follow the implied rule of obtaining consent for blood transfusion without a physician’s order	**(Welfare of others)** Provide beneficial care to patients and families
			Do not obtain consent	**(Rules)** Obtain consent for blood transfusion under the physician’s order **(Legal issues)** Exceeding the scope of work stipulated by law is subject to disciplinary action	
C11	An elderly patient who had refused surgery was rushed to hospital with decreased consciousness and required surgery.	Whether to accept the surrogate decision for consent to surgery by family members	Accept	**(Roles)** Provide life-saving medical care to patients	**(Welfare of others)** Make decisions your family won’t regret **(Appreciation of differences)** Understand the family’s wishes for the patient’s survival
			Do not accept	**(Rights)** Respect the patient’s right to self-determination according to advance directives	
C12	A terminally ill patient who said “Don’t resuscitate” was complaining of suffering.	Whether to suggest to the physician to increase the patient’s sedation	Suggest	**(Rights)** Represent the patient’s distress **(Societal concerns)** Put yourself in patient’s shoes	**(Not hurting)** Relieve patient distress with sedation **(Care of self)** Dispel feeling of remorse for not being able to fulfill the patient’s wishes
			Do not suggest	**(Roles)** Treatment decisions are a physician’s role	
C13	While changing a patient’s diaper with a nursing aide, one patient’s sheets were soiled with feces.	Whether to change sheets with feces on them	Change	**(Roles)** Maintaining the environment and preventing infection by changing sheets **(Obligation and commitments)** Set an example as a leader for other professions **(Societal concerns)** Put yourself in patient’s shoes	**(Welfare of others)** Patients can live comfortably **(Care of self)** Make decisions with confidence as a professional
			Do not change	**(Rules)** Continue changing diapers according to ward routine	
C14	It was decided that surveillance cameras were to be installed in all rooms of the psychiatric ward based on the senior nurse’s opinion.	Whether to require that senior nurses obtain patient consent for the installation of surveillance cameras	Claim	**(Rights)** Should explain the camera to the patient **(Societal concerns)** Put yourself in patient’s shoes	**(Not hurting)** Surveillance is psychologically distressing for patients **(Care of self)** Dispel feeling of powerlessness not being able to say the right thing
			Do not claim	**(Rules)** Someone like the leader of the organization who is not the nursing manager decides things.	
4. Conflict between combinations of justice and care moral categories and care categories					
C15	A patient who had just given birth was admitted for hospitalization for medical treatment and protection	Whether to continue mother-infant separation for the mentally disordered patient immediately after childbirth	Continue		**(Welfare of others)** The patient is mentally stabilized by receiving drug therapy
			Do not continue	**(Rights)** Respect the patient’s right to self-determination	**(Not hurting)** Relieves emotional distress caused by mother-infant separation **(Care of self)** Dispel the feeling of helplessness of not being able to let the patient see the child **(Attachment and connection)** Sympathize with the patient’s distress
C16	A patient on ventilator was physically restrained because they were more likely to self-extubate.	Whether physical restraint to prevent extubation of the tracheal tube should be continued	Continue	**(Rules)** Carry out other duties	**(Welfare of others)** Not self-extubating helps patients get better
			Do not continue		**(Welfare of others)** Attendant for family is a heavy burden **(Care of self)** Dispel self-guilt to using physical restraints **(Not hurting)** Decrease psychological distress caused by physical restraint
C17	Parents of a pediatric patient with half a year to live had considered ventilator use.	Whether to advise parents of the pediatric patient not to use a ventilator	Advice		**(Welfare of others)** Cherish the time the pediatric patient lives **(Appreciation of differences)** Understand the feelings of parents who wish their children to survive
			Do not advice	**(Rights)** Respect the right of children to be free from suffering	**(Not hurting)** Avoid suffering from endotracheal intubation
C18	A terminally ill thyroid cancer patient had not been informed of prognosis.	Whether to give information about the prognosis to the patient	Give		**(Welfare of others)** The patient spends the rest of their lives meaningfully
			Do not give	**(Roles)** Nurses have no authority over patient life expectancy	**(Appreciation of differences)** Understand physicians’ concerns about patients’ fear of death
C19	A patient with a pressure ulcer on the heel had been instructed to wear a foot pump.	Whether to follow the physician’s orders about wearing a foot pump	Follow	**(Roles)** Unable to arbitrarily stop the treatment order of the attending physician	**(Appreciation of differences)** Understand the unpleasant feelings of the attending physician who be objected to the treatment order
			Do not follow		**(Welfare of others)** Avoid worsening the patient’s pressure ulcer
C20	Some colleagues had not been changing diapers or removing phlegm by suction from elderly patients.	Whether to advice the colleagues to change the patient’s diaper changing or removal of phlegm by suction	Advice	**(Roles)** Medical personnel should work as professionals **(Societal concerns)** Put yourself in patient’s shoes	**(Welfare of others)** Deal with the patient if they are in trouble
			Do not advice		**(Appreciation of differences)** Understand the circumstances of colleagues who do not perform their duties
C21	Colleagues objected to administering sedatives to a pulmonary infarction patient who was restless every night.	Whether to administer the patient a sedative against the opinion of colleagues	Administer		**(Appreciation of differences)** Other inpatients are not disturbed
			Do not administer	**(Rules)** Follow a routine that delays the timing of following sedation instructions	**(Welfare of others)** Physician’s order for sedation is inappropriate for the patient’s restlessness **(Appreciation of differences)** Understand colleagues’ suggestions for alternatives to sedation
5. Conflict between justice categories					
C22	The attending physician explained to the patient’s family that the cause of the patient’s death was not his or her fault.	Whether to refute the attending physician, who explains to the patient’s family that they are not at their own fault	Refute	**(Roles)** It is necessary to explain to the family that it was possible to predict and prevent death from suffocation in the patient. **(Legal issues)** We should be justly judged by fact-checking	
			Do not refute	**(Rules)** The attending physician explain to the family **(Obligation and commitments)** Ward manager’s duty is to report the facts to the attending physician.	
C23	A schizophrenic patient with overgrown hair could not get a haircut because he did not have the money for a haircut.	Whether the nurse without a beautician license should cut the patient’s hair	Cut the hair	**(Rights)** Guaranteeing the minimum standard of living as a human being for patients **(Roles)** I learned that body cleanliness is a job that takes the initiative for patients.	
			Do not cut the hair	**(Rules)** I don’t want to be punished, so I follow the hospital’s rule of not cutting patients’ hair. **(Obligation and commitments)** Act in accordance with the duties of the hospital organization	
C24	A physician had obtained consent for cataract surgery from an elderly patient with poor understanding.	Whether to suggest surrogate decisions for the patient’s family to the physician	Suggest	**(Rights)** Patient may lack judgment ability **(Societal concerns)** Put yourself in patient’s shoes	
			Do not suggest	**(Roles)** Physician has obtained patient consent for surgery	
6. Conflict between care categories					
C25	A Parkinson’s patient had difficulty walking alone.	Whether to follow the physician’s orders for gait training	Follow		**(Welfare of others)** Gait training improves activity of daily living in patients **(Appreciation of differences)** Physician’s orders are based on extensive experience
			Do not follow		**(Welfare of others)** Prevent patient falls
7. No conflict with the combination of justice and care moral category					
C26	A physician did not examine the elderly patient who was vomiting after hydrocephalus surgery.	Whether to request the physician to examine vomiting patients	Request	**(Roles)** I need to tell the physician my views about the patient’s examination and treatment **(Societal concerns)** Put yourself in patient’s shoes	**(Welfare of others)** The patient’s pain is gone, and the patient’s life is saved **(Welfare of others)** Patient can return to normal life **(Care of self)** Dispel the feeling of helplessness in not being able to say the right thing
			Do not request		
C27	A nurse reported a lung murmur in a leukemia patient to the attending physician, but he did not examine the patient.	Whether to request the patient be examined by the attending physician	Request	**(Roles)** Don’t overlook the patient’s life-threatening issues	**(Welfare of others)** Early detection and early treatment benefit the patient
			Do not request		
8. No conflict with the care category					
C28	A terminally ill patient with bladder cancer demanded nursing care only from a nurse-in-charge.		Accept		
			Do not accept		**(Welfare of others)** Patient maintains relationships with other staff **(Appreciation of differences)** Incorporate staff input for the patient’s benefit
C29	A pediatric patient’s mother overly interfered with medical procedures performed by nurses.	Whether to respond to the pediatric patient’s mother’s excessive interference	Respond		
			Do not respond		**(Care of self)** Take pride in being a professional **(Care of self)** I should correct the difference in values with the patient’s mother

(　)and Bold = Moral category of each moral perspective according to Chally’s taxonomy

### Conflict between the justice and care moral categories (C1–4)

Moral categories of justice and care supported conflicting options. “Rules,” “roles,” and “obligation and commitments” of justice mainly conflicted with “welfare of others” of care. The moral dilemmas were that “obeying professional authority, organization-specific rules, or obligations conflicted with the pursuit of patient’s well-being.” For example, participants (C4) narrated the dilemma of whether or not to perform the postmortem care on a dead patient with his family. “Rules” supported the option [do not perform the postmortem care with family]:

I have (daily) routines, and I think it would be even better if I had time to close to (the bereaved family) and listen to their stories, but I have to do coolly the exact opposite of what I want to do for them (Justice: rules).

Contrary to the above, “welfare of others” and “care of self” supported the option [perform the postmortem care with family]:

For me personally, I wish to have time to talk to the family and close to them as much as possible… (at the time of postmortem care) I would like the patient’s family to be with the dead patient so that I can put their mind in order. (Care: welfare of others)

I take time to talk with patients’ families as much as possible and value being close to them. I always want to build a relationship of trust with the family, even if it’s just a little. (Care: care of self)

### Conflict between combinations of justice and care moral categories (C5–9)

There were multiple combinations of the justice and care categories, each of which supported conflicting options. Combining these perspectives mainly consisted of the respective combinations of the following categories: “rules” and “welfare of others” (C5, 6, 8, 9); “rules” and “appreciation of differences” (C9); “rights,” “societal concerns,” and “welfare of others” (C6, 7); “rights,” “societal concerns,” “not hurting,” and “care of self” (C5); and “roles” and “appreciation of differences” (C7, 8, 9). These combinations resulted in contradictions and moral dilemmas.

The moral dilemmas were that “observing the organizational-specific rules and wishing for the patient’s recovery and stability conflicted with respect for the patient’s rights and the maintenance of the patient’s comfort” or “understanding the thoughts of the family and colleagues and following professional roles and authority conflicted with maintaining the patient’s comfort and complying with organization-specific rules.” For example, participants (C6) narrated the dilemma of whether or not to suggest increased sedation for the terminally ill patient to physicians. A combination of “rules” and “welfare of others” supported the option [suggest]:

The purpose of sedation is to prevent falls, but in the night shift, I strongly believe that I can’t just be involved with this patient alone in order to carry out my duties… (Justice: rules).

The initial sedation order given to an agitated patient is often aimed at light sedation. It aggravates the patient’s restlessness (Care: welfare of others).

Contrariwise, a combination of “rights,” “societal concerns,” and another “welfare of others” supported the option [do not suggest]:

I wonder if sedation is really a good thing for this patient…how much decision-making rights does the patient have in a family decision… (Justice: rights).

If you think of the patient as my relative, you would not use drugs to put them to sleep (Justice: societal concerns).

In fact, I want the patient to spend the rest of their limited life comfortably. Palliative care should have it. (Care: welfare of others)

### Conflict between the combination of justice and care moral categories and justice categories (C10–14)

In this type, the option supported by the combination of both justice and care categories conflicted with the option supported by another justice category. In the combination of both perspectives, the combinations of “roles” and “welfare of others” (C11, 13), “rights,” “societal concerns,” “not hurting,” and “care of self” (C12, 14), “rules” and “welfare of others” (C10) were mainly used. These combinations were mainly in conflict with “rules” (C10, 13, 14), “rights” (C11), or “roles” (C12) of justice. The moral dilemma included “obeying one’s professional role or authority and wishing for the well-being of the patient or family conflicts with respect for the patient’s rights or adherence to organization-specific rules,” or “respecting the patient’s rights and not to inflict pain on patients conflicts with obeying professional authority or organization-specific rules.” For example, participants (C11) narrated the dilemma of whether or not to accept the surrogate decision for consent to surgery by family members. A combination of “roles,” “welfare of others,” and “appreciation of differences” supported the option [accept the surrogate decision]:

(The patient) has been transported by ambulance, so the medical care that can be provided as a medical staff must be performed, of course, if there is a possibility (of the patient’s recovery) … well… I thought it was a health professional’s role to perform surgery for that purpose, or rather, as a nurse, to provide medical care (to patients) as a merit of medical care. (Justice: roles)

How to explain to the family is important, and if we don’t get involved in making the family’s decisions while considering the background (of the patient and family), (the family) will regret it. (Care: appreciation of differences)

Contrariwise, “rights” supported the option [do not accept the surrogate decision]:

I think it’s better to respect the patient’s intentions. I think that the patient wonders why (the medical staff) doesn’t do what he says. (Justice: rights)

### Conflict between the combination of justice and care moral categories and care categories (C15–21)

In this type, the option supported by the combination of justice and care categories conflicted with the option supported by another care category. In the combination of both perspectives, the combination of “rights” and “not hurting” (C15, 17), “rules” and “welfare of others” (C16, 21), “roles” and “appreciation of differences” (C18, 19), and “roles” and “welfare of others” (C20) were mainly used. These combinations were mainly in conflict with “welfare of others” (C15–19) or “appreciation of differences” of care (C20, 21). The moral dilemma included “respecting the patient’s rights and eliminating the patient’s pain conflicts with valuing the patient’s life” or “adherence of the organizational-specific rules and the patient’s recovery or stability conflicts with not negatively affect family members or other patients.” For example, participants (C17) narrated the dilemma of whether or not to advice parents of the pediatric patient not to use a ventilator. A combination of “rights” and “not hurting” supported the option [do not advice]:

Children’s rights… I thought that endotracheal intubation would be grueling for that child.… (Justice: rights)

I thought that endotracheal intubation is grueling for the child and I shouldn’t do it…I want to quit because it is hard for the child (Care: not hurting).

Contrariwise, “welfare of others” and “appreciation of differences” supported the option [advice not to use a ventilator]:

The child is living life to the fullest…I saw him trying to live…he was unconscious, but I think we shouldn’t give up on his life…maybe. That living time is important to him… (Care: welfare of others).

Parents of dying children want their child to be cared for to the end. I want to accept that feeling. (Care: appreciation of differences)

### Conflict between justice categories (C22–24)

In this type, only the perspective of justice applied, and the justice categories supported conflicting options, mainly between “roles” (C22–24), “rules” (C22, 23), and “rights” (C24). Moral dilemmas involved the conflict between professional roles and organization-specific rules, patient’s rights and organization-specific rules, and patient’s rights and professional roles. For example, participants (C23) narrated the dilemma of whether or not a nurse without a beautician license should cut the hair of the patient. “Rights” and “roles” supported the option [to cut the patient’s hair]:

In various ways, I think there is a minimum standard to guarantee the patient’s life… It is morally problematic that a haircut cannot be done due to money or hospital circumstances (Justice: rights).

I have learned (in professional education) to take the lead in body cleanliness, such as cutting a patient’s growing fingernails or hair (Justice: roles).

Contrariwise, “rules” and “obligation and commitments” supported the option [do not cut the patient’s hair]:

There’s a rule that nurses don’t cut patients’ hair… I’ll be punished when I break staff rules and cut patients’ hair (Justice: rules).

I wanted to cut the patient’s hair, but I thought it was okay for someone else to do it, not me. The care of the patient will be handled by the medical team, so I don’t have to do everything alone (Justice: obligation and commitments).

### Conflict between care categories (C25)

In this type, only the perspective of care applied, and the care categories supported conflicting options. For example, participants (C25) narrated the dilemma of whether or not to follow the physician’s orders for gait training. “Welfare of others” and “appreciation of differences” conflicted with another “welfare of others.” “Welfare of others” and “appreciation of differences” supported the option [follow the physician’s orders]:

I thought that gait training might be necessary in order to maximize the patient’s physical function, although there is a risk of falling (Care: welfare of others).

Since the physician had a long clinical experience, I thought that the judgment is based on experience, but he thought that walking training would be difficult for the patient. As I was so thinking, I gradually came to think of following the order (Care: appreciation of differences).

Contrariwise, another “welfare of others” supported the option [do not follow the physician’s orders]:

As one of the gait trainings, we are planning to incorporate transfer to a chair alone at time of taking medicine into daily life. The risk of falling is so great that it is the only option (Care: welfare of others).

### No conflict with the combination of justice and care moral category (C26, 27)

In this type, a combination of justice and care categories supported one option, but no moral perspective supported the other. For example, participants (C27) narrated the dilemma of whether or not to request the patient’s examination of the attending physician. “Roles” and “welfare of others” supported the option [request the patient’s examination]:

The sooner we know (about a patient’s condition), the sooner we can deal with them (Justice: roles).

I want to think about the patient first, so I thought that the doctor’s work of confirming (the patient’s breathing sound) would lead to saving the patient’s life (Care: welfare of others).

### No conflict with the care category (C28, 29)

In this type, a combination of moral categories of care perspectives supported one option, but no moral perspective supported another. For example, participants (C28) narrated the dilemma of whether or not to accept the request from the patient who wished to receive care limited to the nurse in charge. “Welfare of others” and “appreciation of differences” supported the option [do not accept the request]:

I thought that the patient would probably become isolated if there was no human relationship with staff other than me (Care: welfare of others).

When I care for my patients only from my point of view, I lose the idea that a slightly different direction might yield better results. (Care: appreciation of differences)

## Discussion

### Aspects of complex confrontation between the justice and care perspectives

In this study, the moral conflict showed conflicting aspects from the perspective of justice and care, while the two perspectives themselves are intricately intertwined due to the fact that not only dose each perspective supports conflicting actions, but a single perspective can support conflicting actions these overlaps complicated the moral conflict.

#### Supporting contradictory actions from each justice and care perspective

Because the justice and care perspectives are contradictory in nature, each perspective may support conflicting actions. In this study, among the many cases that included a conflict between the two perspectives, “rules” of the justice perspective and “welfare of others” of the care perspective frequently occurred in each perspective. These categories were considered to be key concepts within the nurses’ justice and care perspectives. Therefore, we will consider the causes of conflict and their solutions centering on two categories.

#### Conflict between “rules” and “welfare of others”

This conflict was attributed to be organizational constraints influencing patient care. In this study, “rules” and “welfare of others” were in conflict with each other and showed distinctive characteristic of the conflict between the justice and care perspectives (C2–4, 6, 9, 10, 13,16). The nurses in this study were concerned with meeting specific patient and family needs while adhering to organization-specific rules. Indeed, best nursing practice for patients and families tends to be hampered by organizational constraints [33–36]. Organizational structures have aspects that affect nurse workload, especially staff shortages, high patient turnover, and administrative tasks that cause excessive workload and difficulty in providing adequate patient care [33]. For example, Japan’s 2018 revision of the medical fee schedules determines nurse staffing based on patient severity [37], and organizations must assess patient care needs using severity and medical and nursing need indicators to determine reimbursement for inpatient wards [38]. However, that assessment does not correctly reflect the severity of inpatients, and it is assumed that Japanese nurses have excessive workloads from inflexible staffing [39, 40]. Healthcare facilities require certain organizational constraints to distribute healthcare resource equally to those in need while ensuring the safety of those within the institution. Since these constraints do not consider the involvement of individual patients or specific situations, conflicts arising from constraints imposed by organizational rules are fundamentally difficult to resolve. However, if organizational factors continue to make the situation undesirable for the patient, the situation may be normalized and the staff may be justified in their actions [41, 42]. Additionally, care staff may omit time-consuming care, citing staff shortage or heavy workloads as an excuse [41, 42]. Such situations should not be overlooked, and organizations have a responsibility to remove and improve barriers to desirable practice for patients [36] and should take steps to lead to conflict mitigations. As one of them, it is suggested to appropriately adjust the staffing according to the workload. Indeed, staffing shortages are caused by sudden events such as rapid deterioration of the clinical condition of patients or an increase in the number of critically ill patients, resulting in an excessive workload [43]. Such workload and staffing mismatches are associated with poor quality of care as well as adverse outcomes of patients [44, 45]. Staffing needs to be adjusted according to workload to enable the provision of care according to individual patient needs, which may be one of the conflict mitigation measures.

#### Conflict between “welfare of others” and “roles”

“Welfare of others” of care was often in conflict with “roles” of justice (C1, 7 ,8, 9, 18, 19). This conflict was attributed to nurses’ lack of authority over clinical decision-making. In this study, much of that authority lay with physicians, team leaders, and nurse managers; nurses were forced to choose between following the decisions of those in authority or advocating for the best interests of individual patients. Nurses tend not to be involved in treatment and care decisions making because of lack of authority of nurses and, as a result, do not act for the ethically desirable decisions for patients [29, 34]. Even if such nurses attempt to be involved in ethical decision-making, they either give up on their involvement because of their experience of getting negative results from moral acts, or justified their non-involvement because of the lack of benefit that their involvement would bring [41]. Such a situation violates the principle of beneficence and nonmaleficence of the healthcare professional. Japanese nurses, the subject of this study, reportedly place more emphasis on the quality of care they provide to patients than do nurses in the United States and China [46]. On the other hand, since nurses’ duties include assisting in medical treatment under the direction of physicians according to Article 5 of the act on Public Health Nurses, Midwives, and Nurses, nurses face greater conflict when the care they wish to provide to patients does not conform to physicians’ treatment policies. Thus, since nurses have different professional roles and duties from physicians and managers, it is difficult to fundamentally resolve this conflict. As one of the measures to mitigate conflict, it is suggested to improve communication within the medical professional team and to develop nurses’ participation in clinical decision-making. Because physicians have to shoulder more legal or professional liability, much of the authority in healthcare falls under their jurisdiction [47]. This has led to an entrenched idea among nonphysician clinicians that they act on the basis of the hierarchical leadership of physicians, which sometimes makes their collaborative approach to leadership difficult for nonphysician clinicians [47]. Communication failure within the interprofessional team is more than just a failure of transfer information and lack of shared understanding; it can lead to delayed in care, medical errors, and poor outcome of patients [48–50]. Additionally, nurses need financial and emotional support from their organizations to participate in patient-related decisions [36]. Their support motivates nurses to act ethically, leading to the provision of quality care to patients.

#### Conflict between “welfare of others” and “rights”

In this study, “welfare of others” of care was also often in conflict with “rights” of justice (C5, 6, 11, 15, 17). This conflict was attributed to the inability of patients with reduced decision-making ability to make decisions about their own well-being. While the nurses in this study believed that recovery of health and sustaining life through treatment would bring the welfare to patients and their families, since the treatment was performed without the patient’s consent and was painful, nurses believed that it was necessary to make decisions on the basis of the patient’s will.

Rights are an important concept in the ethics of justice; however, in medical settings, respecting the patient’s rights is not always considered to lead to patient well-being. For example, in life-threatening emergencies, many patients have reduced decision making capacity, and liberty-restricting measures such as physical restraint and coercive treatment are sometimes prioritized [51]. In such cases, the application of shared decision-making (SDM) is common; however, the choice of SDM surrogate does not always match the patient’s true wishes [52, 53]. In particular, it is recommended that dementia and terminally ill patients express their values and preferences in advance directives to prepare for future disability [54]. However, even with a patient’s advance directive, in practice healthcare professionals do not consistently respect it [55–57], and in immediate and reversible situations, the clinician’s decision making may prevail depending on the patient status [56].

As explained above, if patients have difficulty self-determining or implementing SDM, paternalistic interventions are tolerated in lights of beneficence and nonmaleficence within the ethics of care [58, 59]. However, interventions based on such paternalism are not necessarily affirmed. The patient’s inability to always determine the best health care for him/herself is a situation in which respect for the patient’s autonomy is hindered. The moral dilemma of this study is influenced by Japanese cultural norms. Toda et al. note that Japanese nurses tend to prioritize the wishes of the family over those of the patient, suggesting that Japanese group-centered norms influence decision-making [60]. In addition, Asai et al. pointed out the lack of laws regarding death with dignity and the characteristics of Japanese culture that preserve group harmony, and pointed out the possibility of continuing life-prolonging measures at the request of the family, even when the patient’s death is certain [61]. Such a situation may be also related to the fact that the preparation of advance medical directives is not sufficiently widespread in Japanese medical practice [62]. Furthermore, interventions based on paternalism have various effects on patients. Patients who get coercive treatment may recall such treatment as a negative or positive experience, affecting their quality of life after discharge [51]. Negative recollections lead to a loss of autonomy and dignity of patients, while positive recollections lead patients to appreciate the benefit of the care and acknowledged being treated with respect [51]. Therefore, when it is unavoidable to exercise paternalistic interventions, it is necessary for nurses to treat patients with respect, recognizing the impact of their paternalistic interventions on patients.

### Supporting actions in which a single perspective conflict with itself

In this study, the sometimes self-contradictory nature of the justice and care perspectives complicated not only the conflict between the justice and care perspectives but also moral conflicts.

#### Supporting contradictory actions from justice perspectives

The conflict between moral categories of the justice perspective in this study mainly included the categories of “rules,” “rights,” and “roles” (C5-7, 9–13, 22–24). This conflict was attributed to be dual loyalty. In nurse responses, “rules” indicated organization-specific rules, “roles” indicated professional roles and lack of authority, and “rights” indicated respect for patient self-determination and will. Nurses often had to balance the different interests of patients and their family members or professional duties to a patient and obligations to the interests of a third party [63]. In medical settings, fidelity to patients may conflict with allegiance to colleagues, organizations, or the nation, and two or more roles and associated loyalties and their obligations become incompatible, forcing a moral choice between them [64]. Principles based on the justice perspective in the actual moral conflicts of nurses are the roles and powers inherent in the profession, the rules within the organization, and since they are both professionals and employees, simply choosing one is difficult.

#### Supporting contradictory actions from care perspectives

In this study, conflicts between moral categories of the care perspective included “welfare of others” category. This meant that consideration of well-being according to the needs of patients and their families was essential in nurses’ moral conflicts. The “welfare of others” category often conflicted with “appreciation of differences;” disagreements arose among medical professionals and families about the best care of the patient (C7, 18, 19, 20, 21, 25). This conflict was attributed to differences between caregiver responsibilities. Toronto lists four phases of care – caring about, taking care of, care-giving, and care-receiving – and stated that there is likely to be conflict within each of the phases, and between them [6]. Caregivers often find that many people have their own responsibilities that conflict with each other [6]. In medical settings, those who make up the relationship with the patient are the patient’s family, nurses, and other healthcare professionals, all of whom have different responsibilities in their respective positions. Indeed, in medical settings, family members and medical professionals may make different judgments about the needs of patients, and professionals often have a different opinion [65, 66]. In such a situation, if nurses only recognize the responsibilities of patients, families, and other healthcare professionals, it will be difficult to resolve conflicts, and it will be difficult to make a choice between the various needs that each person perceives.

In addition, in this study, the “welfare of others” category often conflicted with the categories of “not hurting” and “care of self” (C5, 15, 16, 17). Conflicts also arose from contradictory considerations within the “welfare of others” category itself (C6, 16, 25). In particular, in the conflict between “welfare of others” and “care of self” categories, nurses felt guilty to the patient’s suffering due to treatment and procedure. These factors were considered to be due to the multifaceted needs of patients. Nurses are expected to provide holistic support to the patients, but in reality, they sometimes dither over whether to respond to the patient’s physical needs or psychological needs [67]. Healthcare professionals’ prioritization of patient needs in medical settings varies according to the patient’s clinical status and tends to focus on biomedical aspects. Especially in emergency and acute care settings, a dominant biomedical focus by nurses has been identified, with nurses prioritizing the completion of physical care tasks over patients’ psychosocial needs [68, 69]. Among them, nurses’ distress increases when a patient is perceived to be suffering or when relationships between caregivers and distraught family are breaking down [70]. Therefore, nurses feel morally distressed when they are unable to act as advocates for patients and families [71], may be lack of compassion for patients’ suffering, or cause burnout [67, 72]. Patients need to be provided with humanized care, that is, holistic care [73], and it is difficult to prioritize only one aspect of patient needs.

### Significance of the coexistence of justice and care in individuals

#### The relationship between the justice and care perspective-taking and ego development

In this study, most nurses considered their options of action using both justice and care perspectives. The perspective of justice and care is one that everyone has regardless of their occupation [74]. According to the view in moral psychology research, ego development is related to moral reasoning development [75]. Care-based and justice-based reasoning have developmental paths of their own [76]. Care and justice reasoning progress from self-interest concern toward others’ concern by growing capacity to adopt others’ viewpoint, and it may share elements in ego development such as cognitive style, impulse control, and character development [75, 77]. In Juujärvi‘s study, care reasoning was positively related to justice reasoning, suggesting that justice and care complement each other in sophisticated moral reasoning [77]. Therefore, it can be considered that individuals acquire the moral perspective of both justice and care as they achieve moral development along with the ego development.

#### Justice and care in professional ethics

The ethics of justice and care are applicable to ethical decision-making in medical settings and play an important role in healthcare workers’ professional ethics [21, 22, 78]. The code of ethics for nurses explicates respect for human rights, self-determination, and equitable treatment of patients, regardless of their background in accordance with an ethics of justice; it also explicates responsibility to meet patient needs in accordance with an ethics of care [25, 76]. In addition, Green, premised on engendering future humane physician-patient relationship in the future, cites ethics of care as a model for the physician-student relationship in medical education [79]. Indeed, those aspiring for interpersonal care professions such as healthcare and social work make higher quality reasoning from a care perspective than those in other fields (security and business management) [77]. A previous study of physicians, nurses, and medical students found that most people perceive both justice and care in moral conflicts, and some make decisions that combine both perspectives [11, 74, 80, 81]. In particular, responding to the needs of dependent and helpless people is a professional commitment to care for others [82]. Together, the justice and care perspectives provide a rationale for action in terms of providing equitable healthcare to every patient and responding to patient needs.

### Influence on decision-making from a single perspective

Perspectives of justice and care coexist in individuals; however, in some cases only one perspective supports two opposing actions. Thus, distortions can occur when a person’s moral orientation is biased toward either justice or care [7]. First, a moral orientation that only considers the perspective of justice may overlook the needs of patients. The individualistic focus of the ethics of justice leads to an excessively respect for autonomy, ignoring the social conditions necessary for self-determination [7]. In other words, viewing human beings as rational, autonomous individuals, even if they have vulnerabilities and dependencies, leads to a lack of focus on the needs of those who need support [76]. In this study, nurses with only the perspective of justice encountered multiple conflicts based on their professional responsibilities and did not focus on the needs of specific patients (C22–24, Table 2). Justice-oriented or justice-predominant nurses tend to be task-focused based on roles, rules, and obligations. In fact, the lack of equipment and the time-scarce environment due to patient overcrowding in emergency settings caused a loss of dignity for patients requiring specific care, such as terminally ill patients [83]. Real moral conflicts can be observed in patient-specific contexts, and it is not always best evaluated ignoring them and weighing professional obligations.

Focusing only on the perspective of care carries the risk of overlooking the value of autonomy [7]. The relationship between the caregiver and the care-receiver facilitates the creation of a power relationship and risks suppressing the care-receiver’s desires and thoughts [84]. Patients have a sense of dignity and desire to control their lives based on autonomy [85]. In this study, nurses with a care-only perspective focused on the complexity of relationships with the healthcare staff and families and did not focus on patients’ desire (C25, 28, 29, Table 2). Failure to consider the patient’s perspective in the relationship between the caregiver and the care receiver is contrary to the essence of the ethics of care [84]. Such situations lead to disrespect for patient autonomy and risk giving rise to a strong paternalism that places caregivers in strong positions.

### Possibility of conflict resolution

#### Argument from the perspectives of justice and care

To resolve conflicts that are complexly intertwined between perspectives of justice and care, our research suggests the necessity of discussion from both perspectives. Biased discussion from one perspective causes unfavorable distortion. To prevent important oversights in considering ethical issues, discussing them from the perspectives of both justice and care is crucial. Recognizing that justice and care are heterogeneous, care theorists have debated the compatibility of justice and care ethics [5, 7]. Recently, discussing ethics of care has become important when considering ethical issues [14, 15, 81, 86]. The use of ethics of care helps identify and detail serious ethical issues through the interpretation of contextual aspects [86]. Therefore, the ethics of care should be used to resolve complex conflicts involving perspectives of justice and care.

#### Necessity to propose practical measures

The moral conflicts in this research, in which perspectives of justice and care are intricately intertwined, tended to be difficult for those who encountered them to resolve. In this context, nurses tend to behave in conventional patterns of ethical reasoning and practice that follow convention, such as the rules and standards of society, rather than pursuing patient well-being [29, 87]. The conflicts between justice and care in this study arose from organizational constraints related to Japanese cultural norms and laws and the lack of role authority of nurses, and it was considered difficult to fundamentally resolve this issue. However, measures to mitigate conflict in individual situations are necessary. While it is difficult to identify interventions to mitigate complex phenomena involving moral distress, measures that focus on the ethical aspects to be addressed should be implemented [88]. Ethics of care is essential to understanding particular situations and would allow for the consideration of practical measures. As an example, we list the appropriate personnel allocation and the maintenance of the nurse participation system. Improving the organizational environment, such as making rules related to personnel shortages more functional and enabling active discussions within interdisciplinary teams, will help alleviate conflicts. Therefore, it is important to formulate measures to minimize conflicts between the justice and care perspectives as much as possible.

### Limitations

A weakness of this study is sample bias. Since this research is a survey targeting only Japanese nurses, it is an analysis limited to the medical system and healthcare practice of the country. Thus, the moral conflicts obtained in this study may differ from those in other countries. In this respect, the generalizability of the results of this study is limited.

In addition, since this research used a deductive method using Chally’s taxonomy of justice and care, it resulted in the extraction of complex conflicts in which conflicts between perspectives of justice and care and within a single perspective coexist. If an inductive approach had been taken, more essential issues of moral conflict might have been identified.

## Conclusions

The results of this study suggest that nurses make moral judgments based on moral values that reflect a complex interplay of conflicting justice and care perspectives, conflicts within a single perspective, and the complicated overlaps between the perspectives. Organizational constraints, professional authority, and patient characteristics also influenced conflicts between the justice and care perspectives.

In addition, loyalty to patients, organizations, and professions created conflicts within the justice perspective, and multifaceted aspects of care provider responsibilities and patient needs created conflicts within the care perspective. The perspectives of justice and care are important in professional ethics, and it is essential to consider ethical issues from both. Resolving complex moral conflicts is often fundamentally difficult, and it is recommended that an ethic of care be used to “understand” rather than to “resolve” conflicts. It is recommended that both justice and ethics of care, but especially the latter, be used to resolve conflicts.

This study is an attempt to reconcile the moral conflicts faced by healthcare professionals. Its findings have philosophical and scholarly implications because both the justice and care perspectives enrich ethical discussions. Furthermore, there are potential benefits to a healthcare setting if healthcare professionals use the justice and care ethical framework to discuss what is best for patients.

### Electronic supplementary material

Below is the link to the electronic supplementary material.

Supplementary Material 1

Supplementary Material 2

## Data Availability

The datasets used during the current study are available from the corresponding author on reasonable request.

## List of abbreviations

SDM: Shared decision making
