# Correction: Moral conflicts from the justice and care perspectives of Japanese nurses: a qualitative content analysis

**DOI:** 10.1186/s12910-023-00989-8

**Published:** 2023-11-27

**Authors:** Kayoko Tsunematsu, Atsushi Asai, Yasuhiro Kadooka

**Affiliations:** 1 Department of Bioethics, Faculty of Life Sciences, Kumamoto University, 1-1-1 Honjo, Chuo-ku, Kumamoto, 8608556 Japan; 2 Department of Medical Ethics, Tohoku University Graduate School of Medicine, 2-1 Seiryo-machi, Aoba-ku, Sendai, Miyagi, 980-8575 Japan

**Correction to: BMC Medical Ethics (2023) 24:1**

10.1186/s12910-023-00960-7

Following publication of the original article [1], the title “Japanese nurses” needs to be capitalized so the title will as read follows “Moral conflicts from the justice and care perspectives of Japanese nurses: a qualitative content analysis”.

The original article has been corrected.

## References

[1] Tsunematsu K, Asai A, Kadooka Y. Moral conflicts from the justice and care perspectives of japanese nurses: a qualitative content analysis. BMC Med Ethics 2023;24:79. 10.1186/s12910-023-00960-7. 10.1186/s12910-023-00960-7 PMC10552434 37794440

