# Supplementary material for: Moral conflicts from the justice and care perspectives of japanese nurses: a qualitative content analysis
Source: BMC Med Ethics. 2023 Oct 4;24:79. doi: 10.1186/s12910-023-00960-7 (PMC10552434; doi:10.1186/s12910-023-00960-7)
Supplement: Supplementary file 1 — Supplementary Material 1 [file 12910_2023_960_MOESM1_ESM.pdf]

Additional file 1

Real-Life Moral Conflict and Choice Interview

All people have had the experience of being in a situation where they had to make a decision, but weren't sure of what they should do. Would you describe a situation when you faced a moral conflict and you had to make a decision, but weren't sure what you should do?

1. What was the situation? (Be sure you get a full elaboration of the story)
2. What was the conflict for you in that situation? Why was it a conflict?
3. In thinking about what to do, what did you consider? Why? Anything else you considered?
4. What did you decide to do? What happened?
5. Do you think it was the right thing to do? Why/why not?
6. What was at stake for you in this dilemma? What was at stake for others?
   In general, what was at stake?
7. How did you feel about it? How did you feel about it for the other(s) involved?
8. Is there another way to see the problem (other than the way you described it?)
9. When you think back over the conflict you described, do you think you learned anything from it?
10. Do you consider the situation you described a moral problem? Why/why not?
11. What does morality mean to you? What makes something a moral problem for you?
