# Supplementary material for: Moral conflicts from the justice and care perspectives of japanese nurses: a qualitative content analysis
Source: BMC Med Ethics. 2023 Oct 4;24:79. doi: 10.1186/s12910-023-00960-7 (PMC10552434; doi:10.1186/s12910-023-00960-7)
Supplement: Supplementary file 2 — Supplementary Material 2 [file 12910_2023_960_MOESM2_ESM.pdf]

Additional file 2

The interpretive method of data analysis

I. First Reading - Understanding the Story

Briefly Note All Conflicts in the section of the interview entitled “Moral Conflict and Choice” (Please cite page numbers where found).

A. Pleas Make Notes Here on the First Reading – e.g., relationship, general moral language, repeated words and themes, contradictions, and key images and metaphors.

II. Second Reading – Self

A. Self and the Narrative of Action

What actions dose self take in the conflict?

1. Choosing self – Does the narrator see or describe a choice? How is the choice made?
2. What is self describing him/herself as saying and/or doing?
3. What is self thinking or considering or feeling?

B. Self in Relationship

1. What is organizing frame for the relationship(s) described in the conflict?

C. What Is at Stake for Self?

III. Third Reading – Care

A. It the Care Orientation Articulated?

How would you characterize care?

B. If Care It Not (Clearly) Articulated?

What would constitute care in this conflict?

C. Does Self Align with Care? How Do You Know?

Is the alignment explicit or implicit?

IV. Forth Reading – Justice

A. It the Justice Orientation Articulated?

How would you characterize justice?

B. If Justice It Not (Clearly) Articulated?

What would constitute justice in this conflict?

C. Does Self Align with Justice? How Do You Know?

Is the alignment explicit or implicit?
